# Changes in the Sensory Odor Profile during *Chorizo* Maturation and Their Relationship with Volatile Compound Patterns by Partial Least Square Regression (PLS)

**DOI:** 10.3390/foods12050932

**Published:** 2023-02-22

**Authors:** Rosa Pilar Carmona-Escutia, Edith Ponce-Alquicira, María Dolores García-Parra, Socorro Josefina Villanueva-Rodríguez, Héctor B. Escalona-Buendía

**Affiliations:** 1 ESDAI, Universidad Panamericana, Álvaro del Portillo 49, Zapopan 45010, Mexico; 2 Departamento de Biotecnología, Universidad Autónoma Metropolitana, Av. Ferrocarril San Rafael Atlixco 186, Mexico City 09310, Mexico; 3 Unidad de Tecnología Alimentaría, Centro de Investigación y Asistencia en Tecnología y Diseño del Estado de Jalisco, Camino al Arenero No. 1227, El Bajío, Zapopan 45019, Mexico; 4 Unidad de Tecnología Alimentaria, Centro de Investigación y Asistencia en Tecnología y Diseño del Estado de Jalisco, Normalista No. 800, La Normal, Guadalajara 44270, Mexico

**Keywords:** SPME, sensory evaluation, generalized procrustes analysis, multifactor analysis

## Abstract

Odor is one of the most important attributes to determine the overall acceptance of a product. The aim of this investigation is to evaluate the changes in the odor profile and the volatile compounds during thirty-three days of ripening to obtain the pattern of volatile compounds necessary to integrate the odor profile of chorizo (fermented sausage), using Partial Least Squares (PLS). The chili and pork meat odors were predominant during the first five days, vinegar and fermented odors at days twelve and nineteen days, and finally a rancid odor predominated at the end. Only the vinegar, rancid, and fermented odors could be predicted with a good fit model, with the R^2^ coefficient above 0.5, using linear PLS, and the pork meat odor using logarithmic PLS. Each group of volatile compounds interacted in different ways; esters had a positive influence on the vinegar and rancid odors, but a negative on the fermented odor. Some volatile compounds contributed to more than one odor, such as hexanal, ethanol, and ethyl octanoate. This work allowed us to understand the pattern of volatile compounds required to generate some of the specific odors of chorizo; further studies are required to explore the effect of other food components on these patterns of odors.

## 1. Introduction

Fermented meat products, such as chorizo sausage, have diverse odors due to volatile compounds derived from the presence of spices, and those generated during fermentation and ripening. The role of food odors is highly relevant to the overall acceptance of a product [1] and it is a key attribute influencing the amount of food that is ingested, because we perceive the smell (orthonasal) before consumption [2]. Dewik et al. [3] showed that odor and visual texture were the key attributes in deciding the amount of food that is ingested.

The properties of chorizo have been widely studied, such as its sensory properties [4,5,6,7,8], its volatile compound profile during ripening [9,10], and an evaluation of different brands of Pamplona chorizo [11]. Other investigations using chorizo have focused on the effect of ripening time and/or different amounts of some ingredients (fat, starter culture, antioxidants) on volatile compounds, as well as their relationship to the acceptance of the product [12,13] or the effect on the sensory properties [12,13], although sensory tests were only carried out on the final product. Similar studies have been undertaken with other dry-fermented sausages [14,15,16,17]. In some cases, the studies omitted spices to avoid interference in the volatile analysis [18,19]. However, the flavor of these types of products is the result of a complex equilibrium between spices and the variability of volatile compounds derived from different reactions [20]. All of these studies had some limitations, focusing on only a few odors or the global intensity of flavor and/or odor, or the sensory information was focused on preferences and overall acceptability at the end of ripening. Therefore, changes in the odor profile during the whole process of development have been poorly studied in fermented sausages such as chorizo.

Gas Chromatography–Olfactometry (GC-O) has also been used to study fermented sausage aromas [17,21,22]. The technique consists of the elution of a compound mixture through a column in GC coupled with the human olfactory system as a detector. It allows the active odor compounds that are in suprathreshold odor concentrations to be detected, although GC-O detects these active compounds in isolation [23]. It is important to note that the odors we perceive are not due to a single compound; the perception of an odor is the interaction of volatile compound mixtures, with the odorant receptors in a combinatory strategy where one molecule is recognized by more than one receptor, and one receptor recognizes several molecules [24]. Consequently, the odor/aroma we perceive depends on which receptors are activated, so it is also important to understand the pattern of volatile active compounds that activate the receptors that generate the signals for specific odors. To achieve this, multivariate statistics can be used as tools to explore relationships between volatile composition and odor/aroma perception in foods.

Principal Component Analysis (PCA) is the most common multivariate statistical technique used to evaluate the relationship between volatile and sensory information, but it is only an exploratory method, providing an overview of the data. Other modeling techniques must also be explored [25]. Partial least squares (PLS) modeling combines features from PCA and multiple regression to predict a set of dependent variables, the sensory data, from a large set of independent variables, the predictors, which are the volatile compounds [26]. PLS has been applied in other studies on meat products [27,28,29,30], Salami Milano sausage [31], pac choi [32], roasted peanuts [33], and wines [34,35]. To our knowledge, there is no other study that describes the volatile compound pattern which generates the odors in chorizo during the ripening process using this statistical method.

Our aim was to evaluate the changes in the odor profile of chorizo during the maturing process over thirty-three days, and relate this to the changes of volatile compounds, in order to obtain the pattern of compounds that generate each specific odor involved in the odor profile of chorizo, using Partial Least Squares as the multivariate statistical technique.

## 2. Materials and Methods

### 2.1. Sausage Manufacture

Chorizo samples were manufactured in the pilot plant of the Centro de Investigación y Asistencia en Tecnología y Diseño del Estado de Jalisco (CIATEJ). A batch of 12 kg was prepared with 20% pork back fat and 80% lean pork, obtained from a local market in Guadalajara, Jalisco. Lean and back fat were ground through a 5 mm diameter mincing plate (Torrey CI-22-1 L9N2E, Nuevo Leon, Mexico) in the grinder (Torrey Mod. M-22RW, Nuevo Leon, Mexico). Then, they were mixed with the rest of the ingredients in g/kg of mixture: 22 of paprika, 20 of salt, 10 of glucose, 3 of garlic, 1.5 of pepper, 0.5 of sodium ascorbate (all spices were obtained in a local market in Guadalajara, Mexico), and 0.15 of sodium nitrate (Fabpsa^®^, CDMX, Mexico), with 40 mL of water. Starter cultures were not added. The meat mixture was maintained at 4 °C for 24 h and then was stuffed into a synthetic casing with a diameter of 35–38 mm and tied in small pieces of approximately 15 cm, which were then transferred to a dry-ripening chamber where they were kept for 5 days at 6–8 °C and at the relative humidity of the environment (50–80%). Finally, the temperature was increased to 10–12 °C and maintained for 28 days for ripening. A sample of 200–250 g (four pieces of chorizo) was taken for volatile analysis and 500–600 g (eight pieces) for sensory analysis, per day of ripening, at 0, 5, 12, 19, 26, and 33 days (D0, D5, D12, D19, D26, and D33, respectively), and all samples were randomly taken and vacuum-packaged and stored at −20 °C for not more than three months until the respective analyses. Physico-chemical and microbiological analyses of these samples have been previously reported [36].

### 2.2. Sensory Evaluation

The sensory evaluation was carried out by conventional descriptive analysis, focusing only on the odor profile. The panel consisted of seven people (one man and six women between 24 and 45 years old), selected according to their ability to detect and recognize basic tastes and a series of odor compounds (listed in Table 1) selected from previous chorizo, salami, and other sausage studies, and their discrimination ability. For this purpose, a triangle test with two volatile compounds (carvacrol and eugenol) and a duo–trio test with two commercial brands of chorizo were performed.

Commercial and homemade chorizo samples were used to generate the first list of descriptors, then a preliminary list was obtained in two consensus sessions, and the intensity of the preliminary terms in the chorizo samples was evaluated. The final list of descriptors consisted of eight terms: vinegar, fermented, chili, garlic, pepper, greasy, rancid, and pork meat (Table 2). These terms were relevant to describe the chorizo, not redundant or hedonic, and able to discriminate between samples [41].

The training phase consisted of three steps. The first step was recognition and familiarization with different types of spices: onion, garlic, oregano, pepper, cumin, clove, vinegar (acetic acid), and fermented (lactic acid) through a matching test, in which four samples per session were used. The second step aimed at familiarization with simple pork meat mixtures. The first mixtures consisted of minced pork meat and one spice, and then more complex mixtures of minced meat with more than one spice were used. Three mixtures per session were presented to the panelists, and each one was evaluated in duplicate. The third step was evaluating the intensity of the odor terms in various chorizo samples at different times of ripening.

The sensory tests were conducted in a sensory laboratory with individual cabinets kept free from odors. The six samples of chorizo (D0, D5, D12, D19, D26, and D33) were placed in an odorless amber glass bottle (75 mL) covered with aluminum and coded with three digits, presented simultaneously in a randomized order, to be evaluated in duplicate by each judge. The eight odors were included for each replicate (session), but there was a break for each judge after the first four odors. The intensity of each odor was evaluated on a 15 cm non-structured line scale from 0 (not perceived) to 15 (maximum).

### 2.3. Analysis of Volatile Compounds

The volatile extraction was carried out using solid-phase micro-extraction (SPME) in triplicate. Each sample (3 g) was placed inside a 40 mL amber vial, screw-capped with a PTFE/silicone (Supelco, Bellefonte, PA, USA) septum, in a metallic block thermostat at 35 °C for 60 min, based on the methodology reported by Flores & Olivares [42]. After this period of time, a DVB/CAR/PDMS (divinylbenzene/carboxen/polydimethylsiloxane) fiber, film thickness 50/30 μm (Supelco), was placed in the headspace for 120 min at the same temperature. The fiber was conditioned for 60 min at 240 °C prior to extraction.

The methodology for quantification of volatile compounds was adapted from Flores & Olivares [42] and carried out on a Gas Chromatography system GC-2010 (Shimadzu Corporation, Kyoto, Japan) equipped with a flame ionization detector (FID). A split-splitless injection port held at 240 °C was used to thermally desorb the volatile compounds from the SPME fiber onto the front of a DB-624 UI capillary column of 30 m × 0.25 mm, i.d., 1.4 μm film thickness (Agilent J&W, Palo Alto, CA, USA), and nitrogen was used as a carrier gas at a linear velocity of 37 mL/min flow rate. The temperature was 37 °C, isothermal for 13 min, then raised to 110 °C at a rate of 3 °C/min and maintained for 10 min, then raised to 150 at a rate of 3 °C/min, and then 210 °C at a rate of 5 °C/min and held for 10 min. The total run time was 82.67 min. Injector and detector temperatures were both set at 240 °C. The content of each volatile compound was calculated from the FID area and was multiplied by 10^−5^ for easier data management.

Volatile compounds were identified with three complementary approaches. These included mass spectrometry using a GC 6890N (Agilent Technologies, Palo Alto, CA, USA) equipped with a mass selective detector 5975N (Agilent Technologies), using the same column and conditions by quantification section. The mass spectra were obtained by electron impact at 70 eV, acquired over the range m/z 40–500, and compared with the database of the NIST MS library (National Institute of Standards and Technology, Gaithersburg, MD, USA). Additionally, the linear retention indices (LRI) for the compounds were obtained by using the series C5–C18 (Supelco) of alkanes in the CG-FID with the same column and conditions, and compared with the LRI available in the references, which used the same column [12,16,17,18,42,43,44]. Finally, some compounds (these are indicated in Table S2, Supplementary Material) were also compared with the retention time of the authentic standards in CG-FID.

### 2.4. Statistical Analysis

Three-way ANOVA was carried out on the sensory data for each attribute, considering the sample, sessions, and assessor as fixed factors at a 95% significance level in the analysis. Generalized Procrustes Analysis (GPA) was used to evaluate the repeatability, discrimination ability, and panel agreement; parameters were considered to establish that the panel was training. The GPA is widely applied in sensory profiling data and uses translation, rotation/reflection, and isotropic scaling to minimize the effects of the different average scoring positions on a line scale, the interpretation of the attributes, and the different ranges of scoring that assessors use [45].

Means and standard deviations were calculated for the data of volatile compounds. One-way analysis of variance (ANOVA) was carried out to analyze the effect of ripening time. The Tukey multiple range test was applied to compare the significance of means.

Multifactor Factor Analysis (MFA), using ten groups of variables that correspond to nine groups of volatile compounds and sensory data, was applied to explore their evolution during chorizo ripening. Partial Least Square regression (PLS-R) was carried out to predict the model of the pattern of volatile compounds to generate each odor. The fit model to predict each odor by the volatile compounds (R^2^ Y), the capacity of the model to predict the odor (R^2^ X), and the index of quality (Q^2^) were considered to evaluate the quality of each model [46]. The optimum number of the factor was determined by leave-one-out cross-validation (Jackknife-LOO). The volatile compounds with Variable Importance for the Projection (VIP) >1, and standardized coefficients of >0.025, were selected as the most important variables to predict each odor, applying the PLS model. All statistical analyses were performed in XLSTAT (version 2019.2, Addinsoft, Boston, MA, USA).

## 3. Results and Discussion

### 3.1. Sensory Evaluation

**Figure 1:**
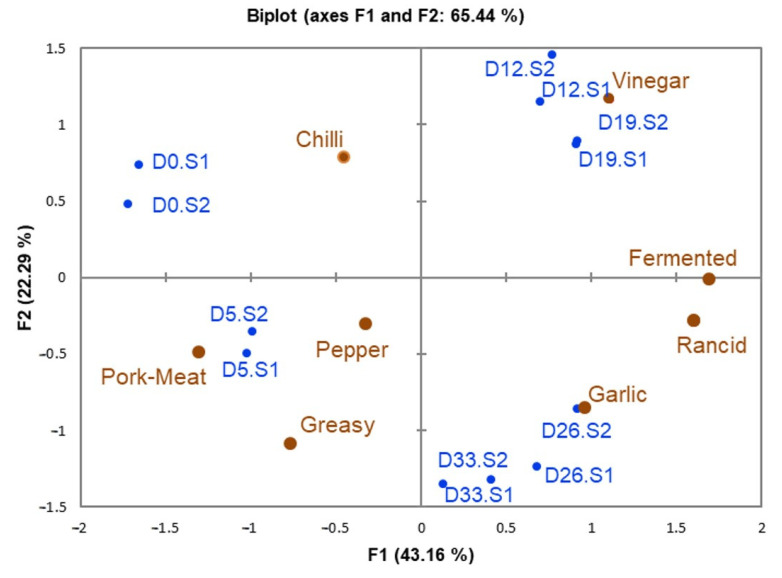
Biplot of Generalized Procrustes Analysis for the six days of ripening of chorizo samples (D0, D5, D12, D19, D26, and D33), and each session (S1 and S2), and the loadings of sensory descriptors.

In general, the ANOVA results of the sensory data (Table S1) showed that factor ripening time (day) had a significant effect (*p* < 0.05) on the eight odors; vinegar, fermented, rancid, greasy, chili pepper, garlic, and pork meat; these results showed that the odor profile changed during ripening. Additionally, the judging factor had a significant effect (*p* < 0.05) on all odors. Even though the judges were trained, differences between them were common, as they used different parts of the scale, but the session factor did not have a significant effect (*p* > 0.05) in any odor, indicating that the judges were consistently able to detect the odor differences at the diverse times of ripening in different sessions [47]. When we analyzed the panel data through GPA, Figure 1, we observed that all judges had the ability to differentiate. Results for the repeatability were similar, as the sample evaluations of sessions one and two were close to each other.

Figure 1 shows the evolution of the odor profile during the ripening of chorizo samples. In general, the GPA explained 65.44% of the variability, and the first GPA dimension (F1) explained 43.16% of the variability. The chorizo at zero and five days of ripening (D0 and D5, respectively) was on the negative side, while the chorizo samples at twelve, nineteen, twenty-six, and thirty-three days of ripening (D12, D19, D26, and D33, respectively) were on the positive side. The beginning of ripening, D0, was characterized by the pork meat odor and chili odor, then these odors decreased during ripening, although the first odor had a slight increase on the final day of ripening, showing similar results to those reported previously [31]. Vinegar, fermented, and rancid odors were detected; these odors were not expected until the final ripening. Nevertheless, some ingredients of chorizo, such as paprika, contain a diversity of organic acids, such as acetic acid, which could be related to the first two odors [10]. The intensity of the garlic odor increased from five days of ripening, but the judges found significant differences (*p* < 0.05) only between D0, with the least intensity, and the chorizo samples at the end of ripening, D26 and D33, with the highest intensity values. This result contrasted with Fernández-Fernandez et al. [4] and Stahnke et al. [31], who found that the garlic odor did not change over time. The garlic odor is mainly associated with sulfur compounds, and previous research has found that the amount of some sulfur compounds, such as allyl methyl sulfide, increased during ripening [48], so this likely explains the increase in the garlic odor.

The second GPA dimension (F2), explaining 22.29% of variability, formed a group with D0, D12, and D19 on the positive side, while the negative side grouped the D5, D26, and D33 samples. The chorizo samples in the middle of ripening, D12 and D19, showed the highest intensity of vinegar and fermented odors that were significantly different (*p* < 0.05), Table S1, from the rest of the ripening days. At the same time, as the vinegar odor increased, we observed an increase in lactic acid bacteria (LAB) growth, data not shown, as reported by Carmona-Escutia et al. [36]. This bacteria group is mainly responsible for carbohydrate fermentation, which generates a diversity of organic acids, such as acetic, propionic, and lactic acid; compounds associated with vinegar and fermented odors [49,50]. Lactobacillus sakei was considered potentially responsible for these sensory characteristics, together with other microorganisms [30,51].

The odor profile in the chorizo samples at the end of the process, D26, showed that the rancid odor had the highest score, with a significant difference (*p* > 0.05), in accordance with other studies on Galician chorizo [4,6,8] and salami [15]. Additionally, in those studies, the global odor intensity was evaluated and shown to decrease during ripening. We observed that although at day thirty-three, D33, fermented and rancid were the predominant odors, both had decreased in intensity compared with D26. Additionally, the intensity of vinegar and chili odors decreased at the end of ripening when compared with D19. According to our results, the global odor is probably integrated vinegar, fermented, rancid, and chili.

### 3.2. Relationship between Volatile Compounds and the Odor Sensory Profile

A total of 120 volatile compounds were extracted by SPME and identified (Table S2). The groups of compounds were twenty-three aldehydes, twenty alcohols, sixteen terpenes, thirteen esters, twelve ketones, eleven alkanes, ten aromatic compounds, eight acids, and seven sulfur compounds.

**Figure 2:**
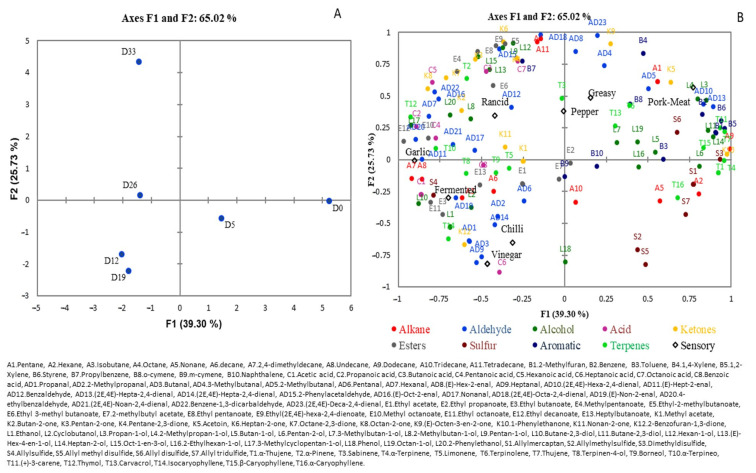
(**A**) Map of days of ripening, D0, D5, D12, D19, D26, and D33. (**B**) Evolution of the groups of volatile compounds and eight odors in chorizo samples using a Multifactor Analysis (MFA).

The MFA, including volatile and sensory data, explained 65.02% of the variability of the data, and this analysis was applied in order to explore the change of different groups of volatile compounds during chorizo ripening, Figure 2. The first MFA dimension (F1) explained 39.30% of the variability, allowing the first days of ripening, D0 and D5, to be differentiated; these were located on the positive side of F1, with the remaining days of ripening, D12, D19, D26, and D33, on the negative side of F1.

Terpenes were the principal groups of volatile compounds related to the pepper, greasy, and pork meat odors at days D0 and D5, Figure 2B. The terpenes were the compounds occurring at the highest amounts at this time of the process, including α-thujene (T1), α-caryophyllene (T16), α-terpinene (T4), thujene (T7), 3-carene (T11), and β-caryophyllene (T15), Table S2. The main sources of these compounds were spices, pepper, and paprika [11,48], which probably explains their relationship with the pepper odor. In general, the major terpenes decreased during ripening (Table S2). Lorenzo, Bedia et al. [15] found similar results and suggested that the volatile compounds from spices, such as pepper and garlic, were lost and/or degraded during ripening. Nevertheless, only T11 and T15 were significant (*p* < 0.05), probably because the amount of spice was not the same in each sample of chorizo analyzed, which generated a large data variation, as is common in flavor analysis in meat products [31].

Sulfur compounds such as allyl mercaptan (S1) and allyl sulfide (S4) showed a slight increase (not significant *p* > 0.5), Table S2, but allyl methyl sulfide (S2) and dimethyl disulfide (S3) decreased significantly (*p* > 0.05). In general, sulfur compounds decreased during ripening, and Gorraiz et al. [52] reported a similar finding for this group of compounds. Some sulfur compounds came from the meat, and these were formed from the degradation of amino acids with sulfur content, such as methionine, cysteine, and cysteine, via Strecker degradation to thiols [53], as well terpenes, like styrene, which came from the meat as a consequence of their presence in animal feedstuff [11], so the relationship between sulfur and terpene compounds with pork meat odor was expected.

Another group of compounds detected at days D0 and D5 were alkanes, such as pentane (A1), hexane (A2), and isobutene (A3), and some alcohols, such as octan-1-ol (L19), butan-1-ol (L5), and hexan-1-ol (L12). Many of these were derived from fatty acids; the source of the fat was the back fat used to make the chorizo, and this possibly explains their relationship with the greasy odor.

Alcohols, aldehydes, and acids were the main groups of volatile compounds present in the middle of the ripening time, D12 and D19, and were related to vinegar, fermented, and chili odors. These were located on the negative side of the second MFA dimension (F2) that explained 25.73% of the variability; Figure 2. During these days, ethanol (L1) was the major alcohol (significant at *p* < 0.05), Table S2; this came from the catabolism of amino acids and carbohydrate fermentation, and was close to the fermented odor, meaning a strong relationship with alcohol existed. Gorraiz et al. [52] found that alcohol also contributes to beef flavor. Other alcohols, such as 2-methylbutan-1-ol (L8) and 2-phenylethanol (L20), were derived from amino acid degradation; these were products of Strecker reactions, associated with the Maillard reaction [20] or caused by bacterial enzymes [16]. Additionally, phenol (L18) occurred in the highest amount at D12 and D19 (*p* < 0.05), then decreased at the end of ripening, probably due to alcohol’s participation in ester formation.

Acetic acid (C1) was also related to the fermented and vinegar odors. C1 was the most abundant acid during the process and came from carbohydrate fermentation, paprika, or compounds from Maillard reactions [11]. It occurred in the highest amount at D19 (*p* < 0.05), then had a slight decrease. Similar results were obtained previously [16,17,18]. Other important acids in these days were propanoic (C2), pentanoic (C4), and heptanoic acids (C6).

The linear saturated aldehydes propanal (AD1), butanal (AD3), and heptanal (AD9) increased significantly (*p* < 0.05) at days D12 and D19, then decreased by the end, D33, and were related to the vinegar and chili odors, Figure 2B. In addition, 2-methylpropanal (AD2) also increased on these days, but not significantly. The branched aldehyde AD2 originated from valine degradation [54]; AD1, AD3, and AD9 were probably formed by an oxidation reaction of their respective alcohols, while AD9 could also have come from autoxidation of fatty acids [53].

The last days of ripening, D26, located on the negative side of F1, and D33, on the positive side of F2, Figure 2A, were related mainly to the rancid odor and various groups of volatile compounds generated by the increase of degradation of amino acids, and oxidation of fatty acids by bacterial enzymes, which related to an increase of molds and LAB at days D26 and D33 [36,51]. The compounds derived from proteolysis or degradation of amino acids, such as 3-methlbutan-1-ol (L7), 2-methylbutan-1-ol (L8), ethyl-3-methyl-butanoate (E6), and benzaldehyde (AD12), showed significant differences (*p* < 0.05), except for the last one; similar findings for these aldehydes were reported previously [20,53]. At D33 of ripening, hexanal (AD7) was the major aldehyde (significant *p* < 0.05); this came from the oxidation of n-6 fatty acids and linoleic and arachidonic acids [52], so has been used as a marker of both lipid oxidation and flavor deterioration [15]. Therefore, the relationship with the rancid odor was expected.

The unbranched alcohols, pentan-1-ol (L9), hexan-1-ol (L12), and octen-1-en-3-ol (L15), increased significantly (*p* < 0.05) at the end of ripening; these came from lipid autooxidation reactions or incomplete β-oxidation [18,19]. Due to their origin, Resconi et al. [55] proposed L12 and other volatile compounds be used as a shelf-life marker in raw beef.

The ketones, pentan-2-one (K3), pentane-2,3-dione (K4), and heptan-2-one (K6), occurred in the highest amounts (significant *p* < 0.05) at D33. These can be produced by lipid oxidation, oxidation of free fatty acids, alkane degradation, or bacterial dehydrogenation of secondary alcohols [53].

Esters were the least abundant group but had an important impact on chorizo odor, probably due to their low threshold, and commonly generate fruit notes [16]. However, in this study, esters were related to the rancid and fermented odors. Esters were formed through the esterification of alcohols and carboxylic acids following microbial esterase activity, mainly attributed to staphylococci, LAB, yeast, and molds [56]. Ethyl butanoate (E3), ethyl pentanoate (E8), methyl octanoate (E10), ethyl octanoate (E11), and ethyl decanoate (E12) significantly (*p* < 0.05) increased their concentrations during ripening, Table S2. The most abundant was ethyl (2E,4E)-hexa-2,4-dienoate, which came from the mixture of sorbic acid and natamycin applied to the sausage casing to prevent surface molds, as previously reported [17].

### 3.3. Pattern of Volatile Compounds of the Specific Odors Using PLS

In order to determine the patterns of volatile compounds, we created two PLS models for each odor, evaluated by sensory analysis: a linear PLS using the raw areas of the volatile data and a log PLS, where a logarithmic transformation of the areas was made. Using the linear PLS model, the results showed the eight odors had a good fit model (R^2^Y), above 0.85, but only vinegar, rancid, fermented, and pork meat models had a good capacity to predict these, because their parameter R^2^ X was above 0.5 [57], and they had a positive fraction of the sensory variable, which can be predicted by components using cross-validation, Q^2^ [50] Table 3.

However, in the linear PLS model, only the volatile compounds with large quantities or with the highest area had a significant weight in the model. According to Chambers & Koppel [58], the logarithmic transformation increases the weight of the volatile compounds found at a lower concentration, which is an important factor to consider, since odor perception not only depends on the concentration of the volatile compound but also on their odor threshold, as well as the interactions that they have with other volatile compounds and other foods components [59]. Therefore, we decided to apply the logarithmic transformation to our data and obtained the logarithmic PLS model.

The results showed that vinegar, fermented, and pork meat odors increased their fitness predictive model using a logarithmic PLS. Lykomitros et al. [33] reported a similar increase in R^2^ X coefficients when they applied logarithmic PLS to predict four flavors of roasted peanuts, suggesting logarithmic transformation was a good choice. Rancid odor had the best model prediction using a linear PLS in terms of the difference in the prediction model of each odor. This can probably be explained as the odor detection or recognition of the major volatile compounds related to fermented, vinegar, and pork meat, which have a logarithmic function, while the compounds related to a rancid odor have a linear function [58]. Then, we determined the volatile compounds that predicted each one of the four odors using the best predictive PLS model.

**Figure 3:**
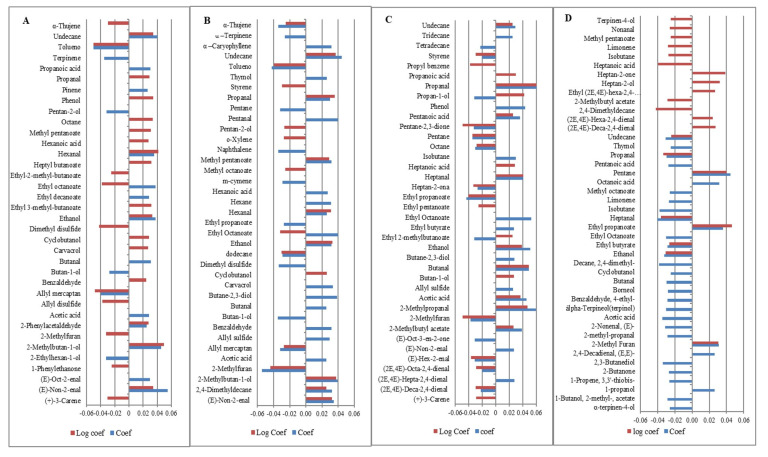
Standardized coefficients of volatile compounds used to predict odors: rancid (**A**), fermented (**B**), vinegar (**C**), and pork meat (**D**).

Vinegar odor was positively related to propanal (solvent), butanal, (pungent), heptanal (fat, rancid), 2-methyl propanal (sour, green), ethanol (sweet), some esters such as ethyl butyrate (apple) and ethyl octanoate (fruit, fat), propanoic acid (pungent, rancid), pentanoic acid (sweet, rancid), heptanoic acid (rancid), and acetic acid (sour) (Figure 3). Often, vinegar odor was only associated with one compound, acetic acid [21,31], but the process by which we perceive one odor, in this case, vinegar, is complex; when we smell food, all volatile compounds reach the nose at the same time, not just one compound, so the vinegar odor was generated by more than one volatile compound. At present, we have no knowledge of another study focused on the vinegar odor in sausages; the closest one was the sour-sock odor investigated by Stahnke et al. [31], which was related to some ketones (butan-2-one, hexan-2-one, heptan-2-one), propanol, sulfur compounds, acetoin, and others. The branched aldehyde, 2-methyl propanal, was the only volatile compound found in common with this study. Compounds negatively related to this odor were styrene (balsamic, gasoline), 3-carene (lemon, resin), hexen-2-enal (apple, green), 2-methylfuran (chocolate), and some alkanes such as octane and pentane (alkene odor, both). These compounds belong to the alkanes and terpenes, and some authors have noted that these groups of volatile compounds are unlikely to contribute to flavor, due to the fact that they have a high threshold [53,60]. Nevertheless, these negatively related compounds also had an impact on vinegar odor, so their presence is probably only the result of creating a synergism, suppression, or increase in other important volatile compounds that are part of the pattern of compounds [38].

Rancid odor was positively related to butanal (pungent), non-2-enal (toast), oct-2-enal (glue), 2-methyl-butan-1-ol (wine, onion), ethanol (sweet), hexanal (grass, fat), propanoic acid (pungent, rancid), acetic acid (sour), 2-phenylacetaldehyde (sweet, green), and some esters, such as ethyl octanoate (fruit, fat) and ethyl decanoate (grape). This odor was negatively related to 2-ethylhexan-1-ol (green), butan-1-ol (medicine), pentan-2-ol (green, plastic), allylmercaptan (garlic), and α-terpinene (woody) Figure 3. Other authors have reported the relationship of rancid flavor with non-2-enal, octanol [38], organic acids such as pentanoic and hexanoic acids, 2-pentyl furan, ethyl octanoate [55], and hexanal [15,38,44,55,60], which is the main volatile compound associated with rancid odor and is strongly related to aroma quality and consumer acceptability [17].

Fermented odor was positively related to non-2-enal (toast), 2-methylbutan-1-ol (wine), ethanol (sweet), propanal (solvent), hexanal (grass, fat), and undecane (alkane), and negatively related to ethyl octanoate (fruit, fat), methyl octanoate (orange), 2-methylfuran (chocolate), allyl mercaptan (garlic), and dodecane (alkane), Figure 3. Some compounds, like 2-methylbutan-1-ol, butanoic acid, pentanoic acid, and ethyl hexanoate, create a fermented odor in cheese [61]. In meat products, Hu et al. [62] found the volatile compounds nonanal, octanal, hexanal, 1-octen-3-ol, heptanol, ethyl hexanoate, ethyl butyrate, ethyl acetate ethyl heptanoate, ethyl octanoate, and methyl hexanoate had an impact on the overall flavor profile of the fermented sausage. Furthermore, Corral et al. [21] found that the esters ethyl 2-methylpropanoate, ethyl-3-methylbutanoate, ethyl butanoate, ethyl pentanoate, and ethyl hexanoate contribute to fermented sausage aroma. Our results also showed that some esters contribute to generating this odor, but indirectly, due to the fact that these had a negative correlation; the presence of esters probably overpowers the fermented odor [57]. Another study found a relationship with 3-methlbutanal, 2-methylbutanal, diacetyl, and 3-methyl butanoic acid [39].

Pork meat odor was positively related to pentane (alkane), 2-methyl furan (chocolate), ethyl propanoate (fruit), heptan-2-one (cheese), and heptan-2-ol (mushroom), and negatively related to 2-methylbuthyl acetate (fruit), ethanol (sweet), ethyl butyrate (apple), heptanoic acid (unpleasant), heptanal (fat, rancid), nonanal (fat, citrus), limonene (citrus), terpinen-4-ol (wood), and undecane (alkane), Figure 3. Furan is the main group of volatile compounds related to meaty flavor, but other authors have found 3-methyl furan, 2-ethyl furan, 2-acetyl furan, and 2-pentyl furan are related to meat odor [21,55]. These are derived from linolenic acid and other n-6-fatty acids [53]. Pavlidis et al. [63] reported that aldehydes such as pentanal, hexanal, decanal, nonanal, and benzaldehyde are characteristic volatile compounds in minced pork meat, explaining why we found the contribution of heptanal and nonanal to the pork meat odor, although these were negatively related. Bueno et al. [64] obtained a negative relationship between meaty odor and aldehyde, saturated and unsaturated. Previous studies have found that ethanol, ethyl octanoate [31], and limonene [21,65] are associated with this odor. Even though the groups of sulfur compounds are important to meaty flavor, in our results, the sulfur compounds did not show an important contribution to pork meat odor, probably due to the methodology that we used to detect the volatile compounds. When we used a linear PLS allyl sulfide we found they did become important to this odor, which was probably because the sulfur compounds are associated mainly with beef flavor [63].

## 4. Conclusions

The odor profile of chorizo changed during ripening; pork meat, pepper, and greasy odors predominated at the beginning of the process, while vinegar and fermented odors predominated in the middle of the ripening period, on days twelve and nineteen, and while rancid was the predominant odor at day thirty-three. However, to determine the best time for ripening, a consumer test should be carried out to relate this odor profile to levels of taste and preference. This is a possible aim of future work.

The use of PLS allowed us to obtain a pattern of volatile compounds to generate the vinegar, fermented, rancid, and pork meat odors. Only these four odors had a good fit model, probably due to the fact that the number of days of chorizo samples ripening used in the study was insufficient since more data is necessary to get better results with this tool. Vinegar, pork meat, and fermented odors improved their fit model when we used a logarithmic transformation in PLS, and rancid odor improved with linear PLS. Odor perception is a complex interaction between diverse groups of volatile compounds; one group of volatile compounds interacts in different ways depending on each odor. For vinegar and rancid odors, the esters had a positive influence, while they had a negative influence on fermented odor. Moreover, some volatile compounds contributed to more than one odor, such as hexanal, ethanol, and ethyl octanoate. In general, this work contributed to the understanding of the pattern of volatile compounds that generate some specific odors of chorizo, but we have not considered how other ingredients could affect the perception of these odors. Therefore, further studies are required where the contribution of some other components, like nonvolatile compounds, are considered.

## Figures and Tables

**Table 1:** List of fourteen odor compounds used during panel selection.

Odor Compound	Brand ^a^	Concentration ^b^ (ppm)	Odor Descriptor
Furfural	SF	90	bread, almond, sweet ^8^
Linalool	SA	3	citrus-like, bergamot-like ^6^
Isoamyl acetate	SA	10	fruity, banana, pear odor ^7^
Eugenol	SA	10	spicy, smoky, clove-like ^7^
Dimethyl sulfide	FK	0.1	Cauliflowers ^1^, cabbage, sulfur ^8^
1-Octen-3-ol	SA	10	earthy, dust, mushroom ^1,2,3,4,5^
Limonene	SA	12	citric, fresh ^1,2,3^
Benzaldehyde	SA	10	bitter almonds ^1^
2,3-Butanedione	FK	0.03	Buttery ^1,4^, cheese ^2,3^
Myrcene	SA	15	hop-like, geranium-like ^6^
Carvacrol	SA	25	medicinal, origanum, herbaceous ^7^
Ethyl butanoate	SF	2	Fruity ^6^
Acetic acid	SF	30000	Vinegar ^4^, pungent, sour ^5^
Hexanal	FK	6	rancid, fresh cut grass ^2,3,4,5^

SF: SAFC, Missouri, USA; SA: Sigma-Aldrich; FK: Fluka, Missouri, USA. a: All Kosher grade; b: Threshold reported by Czerny et al. [37]. ^1^ [38]; ^2^ [39]; ^3^ [22]; ^4^ [17]; ^5^ [40]; ^6^ [37]; ^7^ Flavor-base “http://www.leffingwell.com/flavbase.htm”(accessed on 10 April 2020; ^8^ Flavornet “http://www.flavornet.org/ ”(accessed on 10 April 2020).

**Table 2:** Definition and references used to evaluate the odor intensity.

Odor	Definition	Reference Samples
Greasy	Odor associated with the pork back fat	5 g of pork back fat
Fermented	Odor associated with the lactic acid or cheese	25 mL of 2% (*v*/*v*) lactic acid
Garlic	Odor associated with the garlic powder	5 g of garlic mixture (0.5 g garlic powder/100 g pork meat minced + 50 mL of water)
Vinegar	Odor associated with the acetic acid	25 mL of 0.4% (*v*/*v*) acetic acid
Rancid	Odor associated with the old oil	1 soup spoon rancid olive oil
Chili	Odor associated with the paprika	5 g of chili mixture (1.5 g paprika + 50 mL water/100 g pork meat minced)
Pork meat	Odor associated with the minced pork meat	5 g pork meat minced
Pepper	Odor associated with the pepper powder	5 g of pepper mixture (1 g/100 mL pork meat minced + 50 mL of water)

**Table 3:** Results of quality parameters of the predicted model for the chorizo odors, obtained by linear and logarithmic Partial Least Squares.

Odor	Linear PLS		Logarithmic PLS	
	Q^2^	R^2^	Q^2^	R^2^
Vinegar	0.38	0.69	0.58	0.7
Fermented	0.62	0.54	0.72	0.62
Rancid	0.46	0.76	0.33	0.6
Pork meat	−0.19	0.33	0.65	0.69

Q^2^ Fraction of the sensory variable that can be predicted by components by cross-validation. R^2^ Regression coefficient obtained for the prediction model.

## Data Availability

The data presented in this study are available on request from the corresponding author.

## Supplementary Materials

The following supporting information can be downloaded at: https://www.mdpi.com/article/10.3390/foods12050932/s1, Table S1. Means of the eight odors evaluated by a trained panel during ripening; Table S2. Means and standard deviations of volatile compounds, grouped according to their chemical class, were expressed in UA (1.0 × 10^−5^) on days 0, 5, 12, 19, 26, and 33 of ripening.
