# Supplementary material for: Changes in the Sensory Odor Profile during Chorizo Maturation and Their Relationship with Volatile Compound Patterns by Partial Least Square Regression (PLS)
Source: Foods. 2023 Feb 22;12(5):932. doi: 10.3390/foods12050932 (PMC10000412; doi:10.3390/foods12050932)
Supplement: Supplementary file 1 [file foods-12-00932-s001.zip › foods-2188203-supplementary.pdf]

Supplementary Material

Table S1. Means of the eight odors evaluated by a trained panel during ripening.

	Day of ripening						Factor		
**Odor**	**D0**	**D5**	**D12**	**D19**	**D26**	**D33**	**Rip**	**Jud**	**Ses**
Vinegar	3.6^{cd}	4.3^{bc}	5.9^{a}	6.5^{a}	4.5^{b}	3.5^{d}	***	*	n.s
Fermented	3.9^{c}	4.3^{c}	7.0^{ab}	7.5^{a}	7.4^{ab}	6.5^{b}	***	***	n.s
Pepper	4.9^{ab}	5.5^{a}	5.5^{a}	4.4^{b}	4.9^{ab}	5.4^{a}	**	**	n.s
Chili	6.3^{a}	5.2^{bc}	5.7^{ab}	5.4^{abc}	5.8^{ab}	4.6^{c}	***	**	n.s
Rancid	3.8^{c}	3.8^{c}	6.1^{b}	6.0^{b}	7.0^{a}	5.9^{b}	***	*	n.s
Pork meat	6.1^{a}	4.7^{bc}	3.8^{d}	4.2^{cd}	4.5^{bcd}	5.2^{b}	*	***	n.s
Garlic	2.8^{b}	4.4^{ab}	4.2^{ab}	4.25^{ab}	5.3^{a}	4.6^{a}	**	*	n.s
Greasy	5.4^{b}	6.1^{a}	4.2^{b}	5.2^{b}	5.4^{b}	5.6^{ab}	***	***	n.s

Rip: Effect of ripening, Jud: Effect of judges, Ses: Effect of session.
*significant ($p<0.05$), **significant ($p<0.01$), ***significant ($p<0.001$), n.s, no significance.
^{abc} Different letter in row show significant differences $p<0.05$ by Tukey test during ripening

Table S2. Means and standard deviations of volatile compounds, grouped according to their chemical class, were expressed in UA ($1x10^{-5}$) during 0, 5, 12, 19, 26 and 33 days of ripening.

IVC	LRI	I	Compound[A]	D0	D5	D12	D19	D26	D33	Sig[B]
Alkane										
A1	500	d	Pentane	0.16 ± 0.026^{a}	0.059 ± 0.025^{ab}	0.088 ± 0.102^{ab}	0.032 ± 0.008^{b}	0.056 ± 0.017^{ab}	0.142 ± 0.0115^{ab}	*
A2	600	a	Hexane	0.141 ± 0.120^{a}	0.111 ± 0.157^{a}	0.065 ± 0.013^{a}	0.116 ± 0.006^{a}	0.078 ± 0.054^{a}	0.076 ± 0.047^{a}	n.s
A3	669	e	Isobutane	0.025 ± 0.019^{b}	0.134 ± 0.087^{ab}	0.150 ± 0.036^{a}	0.120 ± 0.025^{ab}	0.059 ± 0.020^{ab}	0.097 ± 0.021^{ab}	*
A4	800	a	Octane	0.193 ± 0.120^{ab}	0.073 ± 0.076^{b}	0.059 ± 0.005^{b}	0.066 ± 0.038^{b}	0.328 ± 0.342^{ab}	0.601 ± 0.283^{a}	*
A5	900	d	Nonane	0.090 ± 0.002^{a}	0.103 ± 0.023^{a}	0.088 ± 0.020^{a}	0.055 ± 0.035^{a}	0.076 ± 0.016^{a}	0.057 ± 0.016^{a}	n.s
A6	1000	a	Decane	0.121 ± 0.047^{a}	0.218 ± 0.064^{a}	0.171 ± 0.043^{a}	0.172 ± 0.028^{a}	0.200 ± 0.041^{a}	0.154 ± 0.032^{a}	n.s
A7	1098	e	2,4-Dimethyldecane	0.089 ± 0.054^{a}	0.116 ± 0.067^{a}	0.232 ± 0.095^{a}	0.189 ± 0.023^{a}	0.186 ± 0.083^{a}	0.175 ± 0.097^{a}	n.s
A8	1100	a	Undecane	0.809 ± 0.497^{bc}	0.622 ± 0.239^{c}	2.476 ± 0.455^{ab}	2.657 ± 0.222^{a}	2.419 ± 0.997^{ab}	1.960 ± 0.916^{abc}	**
A9	1200	a	Dodecane	1.255 ± 0.582^{a}	0.774 ± 0.256^{ab}	0.371 ± 0.036^{b}	0.394 ± 0.035^{b}	0.540 ± 0.255^{ab}	0.510 ± 0.264^{ab}	*
A10	1300	a	Tridecane	0.075 ± 0.038^{a}	0.210 ± 0.195^{a}	0.122 ± 0.030^{a}	0.106 ± 0.029^{a}	0.064 ± 0.011^{a}	0.079 ± 0.029^{a}	n.s
A11	1400	d	Tetradecane	0.019 ± 0012^{b}	0.014 ± 0.006^{b}	0.015 ± 0.000^{b}	0.014 ± 0.003^{b}	0.014 ± 0.004^{b}	0.063 ± 0.031^{a}	**
Aldehydes										
AD1	521	c	Propanal	0.093 ± 0.039^{b}	0.116 ± 0.078^{b}	1.115 ± 0.259^{a}	1.478 ± 0.273^{a}	0.275 ± 0.048^{b}	0.167 ± 0.073^{b}	***
AD2	590	c	2-Methylpropanal	0.505 ± 0.241^{a}	0.366 ± 0.077^{a}	0.714 ± 0.175^{a}	0.933 ± 0.288^{a}	0.373 ± 0.059^{a}	0.530 ± 0.284^{a}	n.s
AD3	619	c	Butanal	0.076 ± 0.017^{c}	0.173 ± 0.118^{bc}	0.415 ± 0.038^{ab}	0.570 ± 0.152^{a}	0.146 ± 0.116^{c}	0.042 ± 0.002^{c}	***
AD4	689	c	3-Methylbutanal	0.062 ± 0.047^{a}	0.023 ± 0.021^{a}	0.027 ± 0.027^{a}	0.014 ± 0.005^{a}	0.068 ± 0.064^{a}	0.067 ± 0.042^{a}	n.s
AD5	692	c	2-Methylbutanal	0.071 ± 0.059^{a}	0.017 ± 0005^{a}	0.020 ± 0.006^{a}	0.020 ± 0.007^{a}	0.055 ± 0.045^{a}	0.053 ± 0.032^{a}	n.s
AD6	734	a	Pentanal	0.218 ± 0.140^{a}	0.129 ± 0.040^{a}	0.174 ± 0.042^{a}	0.318 ± 0.177^{a}	0.278 ± 0.049^{a}	0.176 ± 0.022^{a}	n.s
AD7	836	a	Hexanal	1.375 ± 0.569^{c}	1.691 ± 0.800^{c}	7.882 ± 2.297^{ab}	4.900 ± 1.023^{bc}	9.175 ± 2.101^{a}	8.602 ± 0.954^{ab}	***

IC	LRI	I	Compound[A]	D0	D5	D12	D19	D26	D33	Sig[B]
AD8	904	a	(E)-Hex-2-enal	0.052 ± 0.024 [a]	0.008 ± 0.008 [a]	0.022 ± 0.006 [a]	0.025 ± 0.013 [a]	0.044 ± 0.041 [a]	0.078 ± 0.043 [a]	n.s
AD9	946	c	Heptanal	0.618 ± 0.151[bc]	1.270 ± 0.471 [abc]	1.982 ± 0.242 [a]	1.657 ± 0.171 [ab]	1.481 ± 0.722 [abc]	0.412 ± 0.553 [c]	**
AD10	968	c	(2E,4E)-Hexa-2,4-dienal	0.126 ± 0.084 [a]	0.083 ± 0.049 [a]	0.077 ± 0.042 [a]	0.061 ± 0.012 [a]	0.078 ± 0.027 [a]	0.096 ± 0.035 [a]	n.s
AD11	1007	a	(E)-Hept-2-enal	0.523 ±0.096 [a]	1.064 ± 0.620 [a]	1.400 ± 0.219 [a]	0.968 ± 0.375 [a]	1.303 ± 0.310 [a]	1.133 ± 0.389 [a]	n.s
AD12	1010	a	Benzaldehyde	0.539 ± 0.414 [a]	0.292 ± 0.149 [a]	0.452 ± 0.021 [a]	0.564 ± 0.031 [a]	0.853 ± 0.210 [a]	0.686 ± 0.373 [a]	n.s
AD13	1048	a	(2E,4E)-Hepta-2,4-dienal	0.406 ± 0.415 [a]	0.211 ± 0.185 [a]	0.097 ± 0.019 [a]	0.070 ± 0.023 [a]	0.178 ± 0.133 [a]	0.230 ± 0.178 [a]	n.s
AD14	1064	a	(2E,4E)-Hepta-2,4-dienal	0.550 ± 0.255 [a]	0.713 ± 0.362 [a]	0.976 ± 0.353 [a]	0.646 ± 0.165 [a]	1.001 ± 0.628 [a]	0.468 ± 0.169 [a]	n.s
AD17	1146	c	Nonanal	1.807 ± 1.011 [b]	2.524 ±0.179 [ab]	2.346 ± 0.292 [ab]	2.412 ± 0.245 [ab]	3.665 ±0.391 [a]	2.468 ± 0.411 [ab]	*
AD18	1173	b	(2E,4E)-Octa-2,4-dienal	0.051 ± 0.026 [a]	0.048 ± 0.013 [a]	0.031 ± 0.019 [a]	0.030 ± 0.010 [a]	0.077 ± 0.051 [a]	0.132 ± 0.086 [a]	n.s
AD19	1215	a	(E)-Non-2-enal	0.026 ± 0.012 [a]	0.020 ± 0.011 [a]	0.042 ± 0.020 [a]	0.034 ± 0.013 [a]	0.034 ± 0.010 [a]	0.029 ± 0.007 [a]	n.s
AD20	1238	e	4-Ethylbenzaldehyde	0.008 ± 0.002 [b]	0.274 ± 0.176 [a]	0.301 ± 0.017 [a]	0.247 ± 0.040 [a]	0.327 ± 0.053 [a]	0.318 ± 0.041 [a]	**
AD21	1280	c	(2E,4E)-Nona-2,4-dienal	0.042 ± 0.027 [a]	0.321 ± 0.179 [a]	0.340 ± 0.091 [a]	0.171 ± 0.018 [a]	0.245 ± 0.195 [a]	0.288 ± 0.110 [a]	n.s
AD22	1323	e	Benzene-1,3-dicarbaldehyde	0.019 ± 0.024 [b]	0.132 ± 0.064 [ab]	0.218 ± 0.102 [a]	0.143 ± 0.020 [ab]	0.151 ± 0.043 [ab]	0.304 ± 0.080 [a]	**
AD23	1364	e	(2E,4E)-Deca-2,4-dienal	0.019 ± 0.001 [a]	0.015 ± 0.005 [a]	0.010 ± 0.001 [a]	0.008 ± 0.004 [a]	0.014 ± 0.009 [a]	0.030 ± 0.018 [a]	ns
Alcoholes										
L1	504	b	Ethanol	0.463 ± 0.097[b]	1.288± 0.378[b]	12.617 ± 0.95[a]	14.850 ± 3.954[a]	4.011 ± 0.469[b]	3.936 ± 2.086[b]	***
L2	563	e	Cyclobutanol	0.011 ± 0.009[a]	0.024 ± 0.024 [a]	0.298 ± 0.273 [a]	0.077 ± 0.106 [a]	0.083 ± 0.093 [a]	0.049 ± 0.022 [a]	n.s
L3	608	c	Propan-1-ol	0.344 ± 0.197[a]	0.200 ± 0.166[ab]	0.000 ± 0.0[b]	0.000 ± 0.0[b]	0.158 ± 0.061[ab]	0.186 ± 0.111[ab]	*
L4	683	c	2-Methylpropan-1-ol	0.060 ± 0.052 [a]	0.015 ± 0.015 [a]	0.000 ± 0.0 [a]	0.003 ± 0.001 [a]	0.024 ± 0.017 [a]	0.031 ± 0.027 [a]	n.s
L5	726	a	Butan-1-ol	0.576 ± 0.321 [ab]	1.330 ± 0.855 [a]	0.000 ± 0.0 [b]	0.000 ± 0.0 [b]	0.517 ± 0.207 [ab]	0.234 ± 0.037 [b]	*
L6	754	a	Pentan-2-ol	0.271 ± 0.187 [a]	0.294 ± 221 [a]	0.068 ± 0.032 [a]	0.095 ± 0.019 [a]	0.191 ± 0.098 [a]	0.094 ± 0.042 [a]	n.s

IVC	LRI	I	Compound[A]	D0	D5	D12	D19	D26	D33	Sig[B]
L7	793	a	3-Methylbutan-1-ol	0.987 ± 0.413 [ab]	0.181 ± 0.112 [c]	0.296 ± 0.058 [c]	0.331 ± 0.117 [c]	1.221 ± 0.123 [a]	0.423 ± 0.301 [bc]	***
L8	798	a	2-Methylbutan-1-ol	0.485 ±0.068[b]	0.358 ± 0.088 [b]	0.869 ±0.157 [b]	0.949 ± 0.304 [b]	2.230 ± 0.619 [a]	1.371 ± 0.655 [ab]	**
L9	824	a	Pentan-1-ol	0.500 ±0.254 [b]	0.996 ± 0.173 [b]	0.887 ± 0.222 [b]	0.730 ± 0.112 [b]	0.855 ±0.103 [b]	2.586 ± 0.935 [a]	**
L10	880	a	Butane-2,3-diol	0.628 ± 0.250 [b]	1.224 ± 0.341 [ab]	1.767 ± 0.203 [ab]	2.370 ± 0.201 [a]	1.772 ± 0.605 [ab]	1.454 ± 0.690 [ab]	**
L11	889	c	Butane-2,3-diol	1.642 ± 1.072 [a]	1.624 ± 2.09 [a]	0.988 ± 0.203 [a]	0.957 ± 0.039 [a]	1.257 ± 0.764 [a]	1.233 ± 0.548 [a]	n.s
L12	921	c	Hexan-1-ol	0.535 ± 0.407 [b]	1.251 ± 0.607 [b]	1.307 ± 0.761 [b]	0.618 ± 0.141 [b]	1.186± 0.486 [b]	6.753 ± 3.17 [a]	**
L13	926	c	(E)-Hex-4-en-1-ol	0.119 ± 0.090 [a]	0.152 ± 0.128 [a]	0.188 ± 0.045 [a]	0.098 ± 0.009 [a]	0.314 ± 0.301 [a]	0.312 ± 0.329 [a]	n.s
L14	952	a	Heptan-2-ol	2.017 ± 1.55 [a]	0.226 ± 0.241 [a]	0.095 ± 0.020 [a]	0.098 ± 0.027 [a]	0.109 ± 0.086 [a]	0.484 ± 0.058 [a]	*
L15	1021	a	Oct-1-en-3-ol	1.368 ± 1.02 [a]	2.543 ± 1.277 [a]	2.731 ± 0.778 [a]	1.641 ± 0.351 [a]	3.743 ± 1.545 [a]	5.094 ± 2.643 [a]	n.s
L16	1072	c	2-Ethylhexan-1-ol	0.670 ± 0.275 [a]	1.041 ± 0.599 [a]	0.611 ± 0.102 [a]	0.392 ± 0.109 [a]	0.662 ± 0.326 [a]	0.520 ± 0.237 [a]	n.s
L17	1074	e	3-Methylcyclopentan-1ol	0.800 ± 0.298 [a]	1.192 ± 0.572 [a]	1.540 ± 0.536 [a]	1.305 ± 0.261 [a]	1.340 ± 0.135 [a]	1.581 ± 0.581 [a]	n.s
L18	1106	a	Phenol	0.071 ± 0.015 [b]	0.085 ± 0.010 [b]	0.101 ± 0.034 [b]	0.157 ± 0.010 [a]	0.000 ± 0.00 [b]	0.000 ± 0 [b]	***
L19	1121	a	Octan-1-ol	0.157 ± 0.151 [a]	0.264 ± 0.181 [a]	0.143 ± 0.033 [a]	0.075 ± 0.024 [a]	0.125 ± 0.029 [a]	0.147 ± 0.055 [a]	n.s
L20	1190	a	2-Phenylethanol	0.576 ± 0.218 [a]	0.581 ± 0.125 [a]	0.842 ± 0.375 [a]	0.736 ± 0.135 [a]	0.620 ± 0.192 [a]	0.887 ± 0.086 [a]	n.s
Acids										
C1	710	a	Acetic acid	1.777 ± 0.829 [b]	2.244 ± 0.782 [b]	6.878 ± 2.068 [a]	7.293 ± 1.547 [a]	4.643 ± 2.217 [ab]	4.852 ± 1.796 [ab]	**
C2	809	a	Propanoic acid	0.053 ± 0.041[a]	0.101 ± 0.076 [a]	0.224 ± 0.030 [a]	0.299 ± 0.055 [a]	0.309 ± 0.404 [a]	0.322 ± 0.370 [a]	n.s
C3	894	a	Butanoic acid	0.057 ± 0.027 [a]	0.093 ± 0.056 [a]	0.084 ± 0.003 [a]	0.089 ± 0.014 [a]	0.068 ± 0.027 [a]	0.143 ± 0.130 [a]	n.s
C4	982	c	Pentanoic acid	0.012 ± 0.002 [a]	0.042 ± 0.029 [a]	0.062 ± 0.024 [a]	0.045 ± 0.016 [a]	0.029 ±0.017 [a]	0.060 ± 0.077 [a]	n.s
C5	1062	a	Hexanoic acid	0.022 ± 0.004 [c]	0.277 ± 0.202 [bc]	0.481 ± 0.167 [b]	0.457 ± 0.016 [b]	0.628 ± 0.096 [ab]	0.934 ± 0.159 [a]	***

IVC	LRI	I	Compound[A]	D0	D5	D12	D19	D26	D33	Sig[B]
C6	1180	a	Heptanoic acid	0.793 ± 0.342 [a]	1.265 ± 0.696 [a]	1.491 ± 0.886 [a]	1.457 ± 0.313 [a]	1.301 ± 0.822 [a]	0.503 ± 0.367 [a]	n.s
C7	1264	d	Octanoic acid	0.131 ± 0.069 [a]	0.151 ± 0.055 [a]	0.135 ± 0.077 [a]	0.167 ± 0.084 [a]	0.116 ± 0.031 [a]	0.266 ± 0.046 [a]	n.s
C8	1283	e	Benzoic acid	0.063 ± 0.016 [a]	0.125 ± 0.015 [a]	0.064 ± 0.043 [a]	0.172 ± 0.062 [a]	0.155 ± 0.078 [a]	0.119 ± 0.024 [a]	n.s
<u>*Ketones*</u>										
K1	570	a	Methyl acetate	0.111 ± 0.099 [a]	0.092 ± 0.066 [a]	0.091 ± 0.136 [a]	0.157 ± 0.129 [a]	0.110 ± 0.170 [a]	0.124 ± 0.160 [a]	n.s
K2	632	a	Butan-2-one	0.012 ± 0.007 [a]	0.020 ± 0.007 [a]	0.021 ± 0.008 [a]	0.018 ± 0.001 [a]	0.015 ± 0.002 [a]	0.024 ± 0.020 [a]	n.s
K3	731	c	Pentan-2-one	0.087 ± 0.036 [c]	0.243 ± 0.021 [bc]	0.480 ± 0.065 [bc]	0.504 ± 0.160 [b]	0.427 ± 0.166 [bc]	1.517 ± 0.270 [a]	***
K4	737	a	Pentane-2,3-dione	0.151 ± 0.081 [b]	0.000 ±0.0 [b]	0.053 ± 0.003 [b]	0.245 ± 0.152 [b]	0.175 ± 0.048 [b]	0.569 ± 0.222 [a]	**
K5	784	a	3-Hydroxybutan-2-one (acetoin)	0.357 ± 0.136 [a]	0.242 ± 0.143 [a]	0.154 ± 0.088 [a]	0.148 ± 0.074 [a]	0.306 ± 0.160 [a]	0.297 ± 0.198 [a]	n.s
K6	934	a	Heptan-2-one	0.000 ± 0.0 [b]	0.055 ± 0.020[b]	0.037 ± 0.013 [b]	0.040 ± 0.004 [b]	0.206 ± 0.156 [b]	0.639 ± 0.284 [a]	***
K7	1018	c	Octane-2,3-dione	0.387 ± 0.216 [a]	0.512 ± 0.239 [a]	0.681 ± 0.374 [a]	0.634 ± 0.105 [a]	0.603 ± 0.141 [a]	0.977 ± 0.314 [a]	n.s
K8	1027	a	Octan-2-one	0.445 ± 0.275 [a]	0.729 ± 0.338 [a]	0.811 ± 0.389 [a]	0.791 ± 0.253 [a]	0.893 ± 0.081 [a]	1.107 ± 0.146 [a]	n.s
K9	1088	b	(E)-Oct-3-en-2-one	0.142 ± 0.121 [a]	0.133 ± 0.061 [a]	0.091 ± 0.029 [a]	0.054 ± 0.016 [a]	0.089 ± 0.017 [a]	0.212 ± 0.097 [a]	n.s
K10	1126	b	1-Phenylethanone	0.722 ± 0.496 [a]	0.337 ± 0.262 [a]	0.188 ± 0.034 [a]	0.207 ± 0.030 [a]	0.186 ± 0.077 [a]	0.244 ± 0.056 [a]	n.s
K11	1137	a	Nonan-2-one	0.127 ± 0.052 [a]	0.279 ± 0.039 [a]	0.184 ± 0.031 [a]	0.176 ± 0.022 [a]	0.279 ± 0.051 [a]	0.204 ± 0.115 [a]	n.s
K12	1439	e	2-benzofuran-1,3-dione	0.147 ± 0.106 [a]	0.183 ± 0.132 [a]	0.194 ± 0.017 [a]	0.213 ± 0.048 [a]	0.213 ± 0.156 [a]	0.148 ± 0.064 [a]	n.s
<u>*Ester*</u>										
E1	628	a	Ethyl acetate	0.044 ± 0.038[a]	0.020 ± 0.018 [a]	0.013 ± 0.013 [a]	0.117 ± 0.054 [a]	0.065 ± 0.056 [a]	0.046 ± 0.034 [a]	ns
E2	743	c	Ethyl propanoate	0.071 ± 0.074 [a]	0.177 ± 0.180 [a]	0.028 ± 0.030 [a]	0.137 ± 0.195 [a]	0.114 ± 0.055 [a]	0.096 ± 0.075 [a]	n.s
E3	831	d	Ethyl butanoate	0.254 ± 0.094[b]	0.584 ± 0.242 [ab]	0.756 ± 0.121 [a]	0.640 ± 0.114 [ab]	0.439 ± 0.249 [ab]	0.482 ± 0.071 [ab]	*

IVC	LRI	I	Compound[A]	D0	D5	D12	D19	D26	D33	Sig[B]
E4	855	c	Methyl pentanoate	0.056 ± 0.049 [a]	0.121 ± 0.133 [a]	0.110 ± 0.060 [a]	0.101 ± 0.027 [a]	0.160 ± 0.043 [a]	0.193 ± 0.206 [a]	n.s
E5	872	c	Ethyl 2-methylbutanoate	0.000 ± 0.0 [b]	0.000 ±0.0 [b]	0.000 ± 0.0 [b]	0.000 ± 0.0 [b]	0.042 ± 0.008 [ab]	0.084 ± 0.051 [a]	**
E6	876	c	Ethyl 3-methyl butanoate	0.010 ± 0.001 [b]	0.014 ± 0.006 [b]	0.017 ± 0.003 [b]	0.022 ± 0.005 [b]	0.138 ± 0.029 [a]	0.100 ± 0.038 [a]	***
E7	909	c	2-Methylbutyl acetate	0.076 ± 0.049 [a]	1.040 ± 0.792 [a]	0.450 ± 0.046 [a]	0.292 ± 0.216 [a]	0.133 ± 0.016 [a]	0.312 ± 0.255 [a]	n.s
E8	932	c	Ethyl pentanoate	0.000 ± 0.0 [c]	0.019 ± 0.004 [bc]	0.027 ± 0.019 [bc]	0.016 ± 0.005 [bc]	0.055 ± 0.012 [b]	0.107 ± 0.033 [a]	***
E9	1140	e	Ethyl (2E,4E)-hexa-2,4-dienoate	0.000 ± 0.0 [b]	0.056 ± 0.039 [b]	0.038 ± 0.013 [b]	0.128 ± 0.016 [b]	0.424 ± 0.088 [ab]	0.977 ± 0.596 [a]	**
E10	1154	c	Methyl octanoate	0.000 ±0.0 [b]	0.317 ± 0.120 [a]	0.308 ± 0.075 [a]	0.229 ± 0.035 [a]	0.331 ± 0.098 [a]	0.324 ± 0.034 [a]	**
E11	1224	b	Ethyl octanoate	0.000 ± 0.0 [c]	0.000 ± 0.0 [c]	0.200 ± 0.007 [a]	0.262 ± 0.033 [a]	0.121 ± 0.042 [b]	0.112 ± 0.042 [b]	***
E12	1423	d	Ethyl decanoate	0.000 ± 0.0 [b]	0.052 ± 0.016 [b]	0.177 ± 0.055 [a]	0.173 ± 0.057 [a]	0.178 ± 0.061 [a]	0.188 ± 0.032 [a]	***
E13	1450	e	Heptyl butanoate	0.218 ±0.042 [a]	0.316 ± 0.287 [a]	0.303 ± 0.077 [a]	0.311 ± 0.088 [a]	0.460 ± 0.069 [a]	0.259 ± 0.027 [a]	n.s
Sulfur										
S1	588	e	Prop-2-ene-1-thiol (Allyl mercaptan)	1.277 ± 0.826 [ab]	1.787 ± 0.434[a]	0.203 ± 0.142[bc]	0.382 ± 0.279[bc]	0.167 ± 0.001[c]	0.144 ± 0.049[c]	**
S2	714	c	3-methylsulfanylprop-1-ene (Allyl methyl sulfide)	2.281 ± 1.099[a]	1.268 ± 0.410 [a]	2.209 ± 0.537 [a]	1.513 ± 0.699 [a]	1.031 ± 0.785 [a]	0.423 ± 0.203 [a]	*
S3	772	c	(methyldisulfanyl)methane (Dimethyl disulfide)	0.984 ± 0.836 [a]	0.180 ± 0.085 [a]	0.071 ± 0.025 [a]	0.106 ± 0.019 [a]	0.056 ± 0.008 [a]	0.113 ± 0.036 [a]	*
S4	887	c	3-prop-2-enylsulfanylprop-1-ene (Allyl sulfide)	0.656 ± 0.142 [a]	1.020 ± 0.019 [a]	1.080 ± 0.199 [a]	1.491 ± 0.140 [a]	1.446 ± 0.452 [a]	1.033 ± 0.949 [a]	n.s
S5	952	e	3-(methyldisulfanyl)prop-1-ene (Allyl methyl disulfide)	21.32 ± 9.695 [a]	19.025 ± 2.023 [a]	17.810 ± 2.84 [a]	19.597 ± 0.192 [a]	18.857 ± 3.47 [a]	10.709 ± 2.70 [a]	n.s
S6	1115	c	3-(prop-2-enyldisulfanyl)prop-1-ene (Allyl disulfide)	17.749±11.05[a]	10.361±5.94[a]	11.298±2.66[a]	12.881±1.82[a]	9.858±4.653[a]	13.726±6.71[a]	n.s

IVC	LRI	I	Compound[A]	D0	D5	D12	D19	D26	D33	Sig[B]
S7	1346	e	3-(prop-2-enyltrisulfanyl)prop-1-ene (Allyl trisulfide)	0.529 ± 0.331 [a]	0.348 ± 0.108 [a]	0.277 ± 0.092 [a]	0.421 ± 0.177 [a]	0.373 ± 0.066 [a]	0.237 ± 0.094 [a]	n.s
Aromatic Group										
B1	614	c	2-Methylfuran	0.367 ± 0.095[a]	0.310 ± 0.023[ab]	0.095 ± 0.034[c]	0.039 ± 0.016[c]	0.067 ± 0.054[c]	0.175 ± 0.086[bc]	***
B2	677	c	Benzene	0.574 ± 0.420[a]	0.130 ± 0.136 [a]	0.061 ± 0.011 [a]	0.079 ± 0.030 [a]	0.219 ± 0.128 [a]	0.255 ± 0.183 [a]	n.s
B3	790	c	Toluene	1.400 ± 0.151 [ab]	2.270 ± 1.175 [a]	0.781 ± 0.118 [ab]	0.800 ± 0.242 [ab]	0.378 ± 0.063 [b]	1.007 ± 0.707 [ab]	*
B4	892	c	*1,4*-Xylene	0.314 ± 0.216 [a]	0.242 ± 0.195 [a]	0.172 ± 0.031 [a]	0.152 ± 0.003 [a]	0.171 ± 0.098 [a]	0.369 ± 0.334 [a]	n.s
B5	915	e	*1,2*-Xylene	0.495 ± 0.425 [a]	0.202 ± 0.252 [a]	0.020 ± 0.00 4 [a]	0.018 ± 0.006 [a]	0.088 ± 0.123 [a]	0.159 ± 0.253 [a]	n.s
B6	916	c	Styrene	0.278 ± 0.196 [a]	0.191 ± 0.135 [a]	0.031 ± 0.005 [a]	0.030 ± 0.006 [a]	0.077 ± 0.061 [a]	0.131 ± 0.155 [a]	n.s
B7	972	c	Propyl benzene	0.023 ± 0.018 [a]	0.025 ± 0.022 [a]	0.021 ± 0.014 [a]	0.015 ± 0.021 [a]	0.052 ± 0.069 [a]	0.050 ± 0.013 [a]	n.s
B8	1039	e	1-methyl-2-propan-2-ylbenzene (*o*-cymene)	1.145 ± 0.417 [a]	1.322 ± 1.112 [a]	0.534 ± 0.092 [a]	0.635 ± 0.083 [a]	1.453 ± 1.391 [a]	1.089 ± 1.023 [a]	n.s
B9	1184	e	1-methyl-3-propan-2-ylbenzene (*m*-cymene)	0.089 ± 0.0105 [a]	0.538 ± 0.536 [a]	0.200 ± 0.028 [a]	0.144 ± 0.040 [a]	0.255 ± 0.016 [a]	0.156 ± 0.018 [a]	n.s
B10	1232	e	Naphthalene	0.069 ± 0.024 [a]	0.114 ± 0.046 [a]	0.086 ± 0.010 [a]	0.061 ± 0.004 [a]	0.053 ± 0.016 [a]	0.075 ± 0.028 [a]	n.s
Terpens										
T1	936	c	4-methyl-1-propan-2-ylbicyclo[3.1.0]hex-3-ene (α-Thujene)	0.443 ± 0.457 [a]	0.421 ± 0.423 [a]	0.154 ± 0.020 [a]	0.148 ± 0.032 [a]	0.185 ± 0.040 [a]	0.148 ± 0.042 [a]	n.s
T2	939	a	4,6,6-trimethylbicyclo[3.1.1]hept-3-ene (α –Pinene)	0.266 ± 0.232 [a]	1.150 ± 0.928 [a]	0.780 ± 0.069 [a]	0.764 ± 0.109 [a]	1.078 ± 0.613 [a]	1.539 ± 1.487 [a]	n.s
T3	985	e	4-methylidene-1-propan-2-ylbicyclo[3.1.0]hexane (Sabinene)	0.064 ± 0.013 [a]	0.088 ± 0.014 [a]	0.064 ± 0.022 [a]	0.071 ± 0.012 [a]	0.057 ± 0.022 [a]	0.088 ± 0.026 [a]	n.s

IVC	LRI	I	Compound[A]	D0	D5	D12	D19	D26	D33	Sig[B]
T4	1025	c	1-methyl-4-propan-2-ylcyclohexa-1,3-diene (α –Terpinene)	0.658 ± 0.200 [a]	0.513 ± 0.063 [a]	0.374 ± 0.107 [a]	0.391 ± 0.034 [a]	0.477 ± 0.208 [a]	0.397 ± 0.148 [a]	n.s
T5	1045	b	(4R)-1-methyl-4-prop-1-en-2-ylcyclohexene (Limonene)	0.275 ± 0.132 [c]	0.811 ± 0.114 [a]	0.559 ± 0.142 [ab]	0.463 ± 0.033 [bc]	0.522 ± 0.012 [bc]	0.510 ± 0.048 [bc]	**
T6	1068	c	1-methyl-4-propan-2-ylidenecyclohexene (Terpinolene)	0.901 ± 0.088 [a]	1.094 ± 0.374 [a]	0.785 ± 0.110 [a]	0.805 ± 0.032 [a]	0.879 ± 0.029 [a]	0.962 ± 0.433 [a]	n.s
T7	1160	b	4-methyl-1-propan-2-ylbicyclo[3.1.0]hex-2-ene (Thujene)	1.672 ± 0.596 [a]	1.239 ± 0.328 [a]	0.942 ± 0.212 [a]	0.897 ± 0.105 [a]	1.106 ± 0.326 [a]	1.120 ± 0.220 [a]	n.s
T8	1223	b	4-methyl-1-propan-2-ylcyclohex-3-en-1-ol (Terpinen-4-ol)	0.778 ± 0.367 [b]	1.696 ± 0.098 [a]	1.445 ± 0.236 [a]	1.260 ± 0.040 [ab]	1.533 ± 0.191 [a]	1.282 ± 0.147 [ab]	**
T9	1228	e	4,7,7-trimethylbicyclo[2.2.1]heptan-3-ol (Borneol)	0.181 ±0.080 [a]	0.367 ± 0.076 [a]	0.320 ± 0.051 [a]	0.236 ± 0.024 [a]	0.289 ± 0.099 [a]	0.266 ± 0.066 [a]	n.s
T10	1244	e	2-(4-methylcyclohex-3-enyl)propan-2-ol (α –Terpineol)	0.244 ± 0.07 [c]	0.707 ± 0.058 [ab]	0.688 ± 0.101 [ab]	0.545 ± 0.079 [b]	0.766 ± 0.043 [a]	0.655 ± 0.059 [ab]	***
T11	1362	e	4,7,7-trimethylbicyclo[4.1.0]hept-3-ene ((+)-3-Carene)	0.623 ± 0.180 [a]	0.504 ± 0.130 [ab]	0.316 ± 0.057[b]	0.381 ± 0.080 [ab]	0.438 ± 0.027 [ab]	0.442 ± 0.045 [ab]	*
T12	1384	b	5-methyl-2-propan-2-ylphenol (Thymol)	0.049 ± 0.006 [b]	1.094 ± 0.181 [ab]	1.622 ± 0.433 [ab]	1.438 ± 0.118 [ab]	1.923 ± 1.330 [a]	2.096 ± 0.646 [a]	*
T13	1396	b	2-methyl-5-propan-2-ylphenol (Carvacrol)	0.526 ± 0.272 [a]	0.204 ± 0.039 [a]	0.307 ± 0.095 [a]	0.318 ± 0.030 [a]	0.525 ± 0.052 [a]	0.383 ± 0.092 [a]	n.s
T14	1445	e	(1R,4Z,9S)-4,11,11-trimethyl-8-methylidenebicyclo[7.2.0]undec-4-ene(Isocaryophillene)	0.663 ± 0.236 [a]	0.717 ± 0.403 [a]	0.917 ± 0.103 [a]	1.005 ± 0.178 [a]	0.929 ± 0.571 [a]	0.688 ± 0.370 [a]	n.s

IVC	LRI	I	Compound[A]	D0	D5	D12	D19	D26	D33	Sig
T15	1457	e	(1R,4E,9S)-4,11,11-trimethyl-8-methylidenebicyclo[7.2.0]undec-4-ene (β-Caryophyllene)	29.15 ± 14.85 [a]	16.572 ± 4.243 [a]	10.394 ± 0.11 [a]	12.970 ± 3.241 [a]	21.394 ± 9.53 [a]	13.916 ± 1.67 [a]	n.s
T16	1497	e	(1E,4E,8E)-2,6,6,9-tetramethylcycloundeca-1,4,8-triene (α –Caryophyllene)	1.497 ± 0.662 [a]	0.744 ± 0.484 [a]	0.431 ± 0.164 [a]	1.136 ± 0.393 [a]	0.954 ± 0.398 [a]	0.495 ± 0.070 [a]	n.s

IVC: Identification of volatile compounds

LRI: Linear Retention Index

I: Volatile compounds identified by: (a) Mass spectrum, LRI of authentical standard and LRI of bibliography; (b) Mass spectrum and LRI of authentical standard; (c) Mass spectrum and LRI of bibliography; (d) LRI of authentical standard and bibliography; (e) Mass spectrum.

[A]IUPAC names

Sig: Significance of the effect of ripening time: *significant ($p<0.05$), **significant ($p<0.01$), ***Significant ($p<0.001$), n.s, no significance.

[abc] Different letter in row show significant differences $p<0.05$ by Tukey test.
